# An integrated primary care workforce planning toolkit at the regional level (part 2): quantitative tools compiled for decision-makers in Toronto, Canada

**DOI:** 10.1186/s12960-021-00595-y

**Published:** 2021-07-21

**Authors:** Sarah Simkin, Caroline Chamberland-Rowe, Ivy Lynn Bourgeault

**Affiliations:** University of Ottawa and Canadian Health Workforce Network, Ottawa, Canada

**Keywords:** Integrated health workforce planning, Primary care, Population health needs, Regional planning, Multi-professional, Service-focused, Practice patterns, Population mobility

## Abstract

**Background:**

Health workforce planning capability at a regional level is increasingly necessary to ensure that the healthcare needs of defined local populations can be met by the health workforce. In 2016, a regional health authority in Toronto, Canada, identified a need for more robust health workforce planning infrastructure and processes. The goal of this project was to develop an evidence-informed toolkit for integrated, multi-professional, needs-based primary care workforce planning for the region. This article presents the quantitative component of the workforce planning toolkit and describes the process followed to develop this tool.

**Methods:**

We conducted an environmental scan to identify datasets addressing population health need and profession-specific health workforce supply that could contribute to quantitative health workforce modelling. We assessed these sources of data for comprehensiveness, quality, and availability. We also developed a quantitative health workforce planning model to assess the alignment of regional service *requirements* with the service *capacity* of the workforce.

**Results:**

The quantitative model developed as part of the toolkit includes components relating to both population health need and health workforce supply. Different modules were developed to capture the information and address local issues impacting delivery and planning of primary care health services in Toronto.

**Conclusions:**

A quantitative health workforce planning model is a necessary component of any health workforce planning toolkit. In combination with qualitative tools, it supports integrated, multi-professional, needs-based primary care workforce planning. This type of planning presents an opportunity to address inequities in access and outcome for regional populations.

## Background

The goal of health workforce planning is to ensure alignment between population health needs and the capacity of the health workforce to meet those needs. While it is typical for workforce planning models to operate at national, provincial or state scales, regional workforce planning—at sub-national and sub-provincial planning geographies—helps to focus on the more proximate healthcare needs of cities, communities, and neighbourhoods. Planning at a regional level can promote both accuracy and equity by helping to ensure that the healthcare needs of defined local populations can be met by the locally available health workforce. Unmet regional needs have highlighted the inadequacies of planning at broader scales and have created an impetus for regional planning in order to guide efficient deployment of resources—health workforce and otherwise—where they are most needed.

In Canada, a federated system with universal health coverage, healthcare largely falls under provincial jurisdiction. Each province organizes, funds, and administers the provision of healthcare services. In Ontario, Canada’s most populous province, regional health authorities have an administrative role in diverse healthcare services including hospitals, Community Health Centres, long-term care homes, mental health and addiction agencies, and community support service agencies. In 2016, the *Patients First Act* (Bill 41) added health workforce planning to the mandate of these regional health authorities and they became involved in administering the organization and integration of primary care health services.

**Fig. 1:**
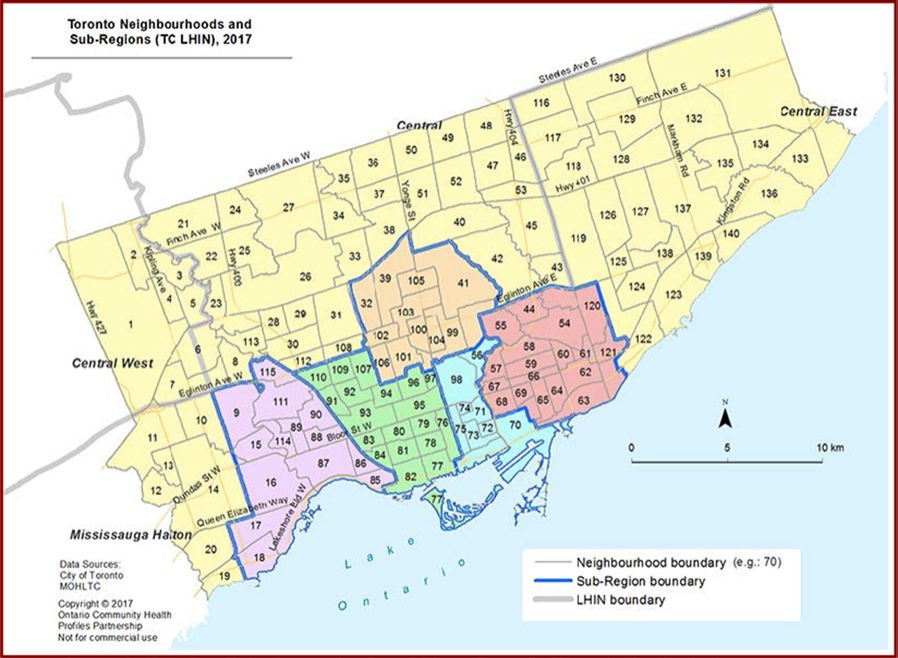
City of Toronto neighbourhoods and central sub-regions

In Toronto, the regional health authority responsible for administering healthcare services for the 2.7 million residents of the city is called the Toronto Region (formerly the Toronto Central Local Health Integration Network (LHIN)). The Toronto Region plans, integrates, and funds health services—including primary healthcare—at a local level. The City of Toronto is divided into 140 neighbourhoods that were defined to help government and community organizations with local planning. Neighbourhoods are also grouped together into larger planning areas (sub-regions). Toronto neighbourhoods and the five central sub-regions are shown in Fig. 1.

Rapidly changing demographics and disparities in access to integrated primary care between sub-regions provided the impetus for the development of a comprehensive Primary Care Strategy. This strategy, which was developed with patient and provider input, aims to improve patient access to care, service integration, and system efficiency. The Toronto Region recognized that successful implementation of this strategy was contingent upon adequately planning for the health workforce needs of the future and that a robust, regional-level planning framework was necessary.

Accordingly, our team at the Canadian Health Workforce Network (CHWN) was contracted to co-develop an evidence-informed toolkit for integrated, population needs-based, multi-professional primary care workforce planning in the City of Toronto. Guided by an overarching framework and set of key principles outlined in an introductory commentary by Bourgeault et al. [1], the toolkit developed is a collection of fit-for-purpose qualitative, descriptive and quantitative processes to guide and support the Toronto Region in conducting health workforce planning activities.

**Fig. 2:**
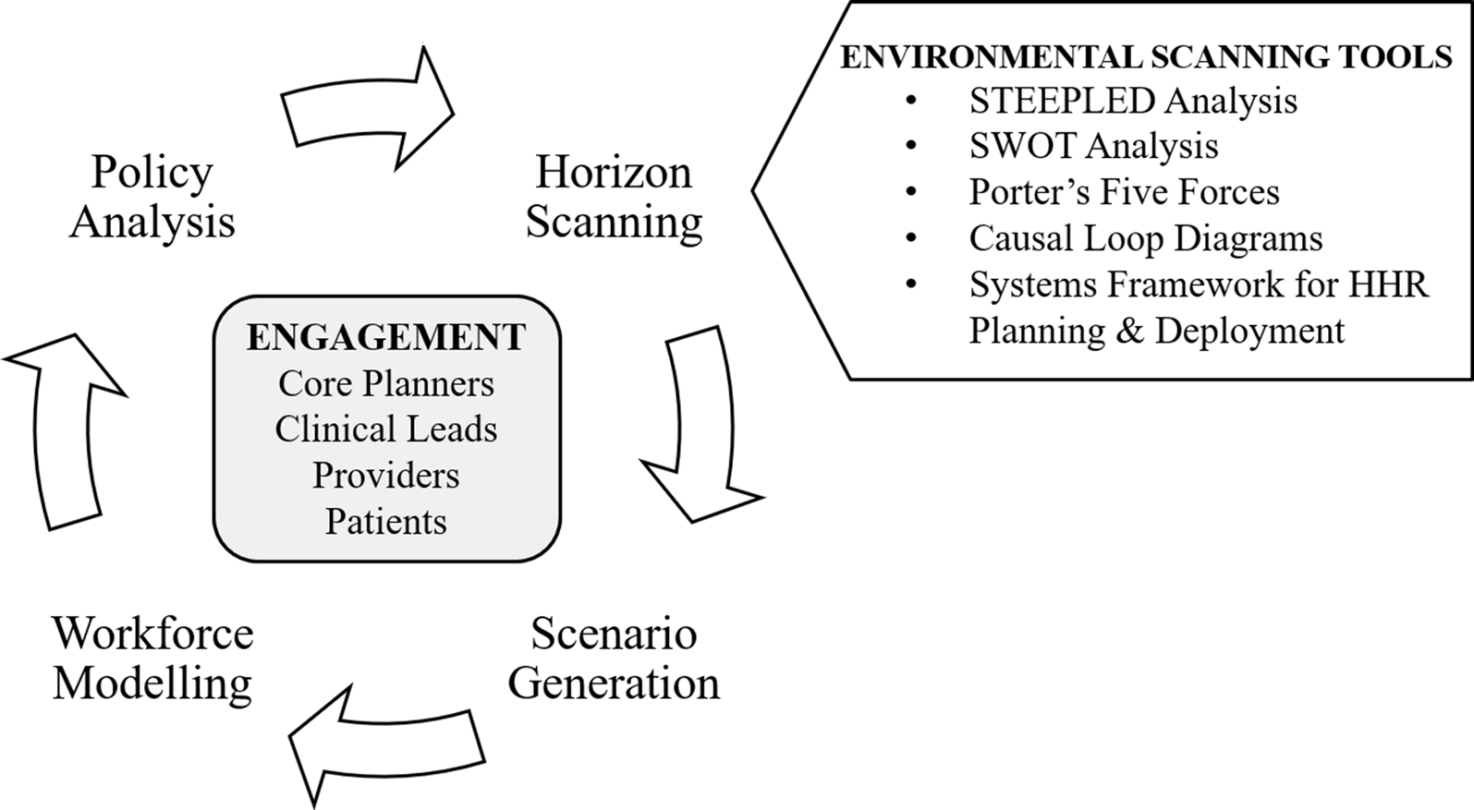
Cyclical health workforce planning process

A comprehensive review of existing models and methods in health workforce planning synthesized leading practices in the development of a workforce planning process, assessing their applicability to the planning needs of the Toronto Region before adapting and integrating relevant approaches into the planning toolkit (see part 1 by Chamberland-Rowe et al. [2]). Our intention was to acknowledge and address key challenges faced by our regional partners, and to tailor the approach to the local planning needs of the Toronto Region. As a result, the toolkit presents a fit-for-purpose planning process that mobilizes the best and most relevant tools available to allow for integrated, multi-professional, needs-based primary care workforce planning at a regional level. The cyclical planning cycle includes four phases—horizon scanning, scenario generation, workforce modelling, and policy analysis—that reflect the iterative and cyclical nature of planning (Fig. 2).

This article is one of two that describe the co-development of an evidence-informed, fit-for-purpose, toolkit-based approach to primary care health workforce planning. The first paper (part 1) [2] outlines the methods and findings from a review of leading practices in health workforce planning and describes the qualitative tools included in the toolkit. This paper (part 2) describes the process we followed to identify the data necessary to facilitate quantitative health workforce planning and introduces the fit-for-purpose modular quantitative model—one element of the toolkit—that we developed to allow the Toronto Region to conduct needs-based planning.

## Methods

We adopted a hybrid set of methodologies, refined at each stage, to produce a fit-for-purpose end-product. Using an approach informed by a participatory action research framework [3], we collaborated closely throughout the project with our primary partners: Toronto Region leadership and analytics staff, primary care physician community leaders, and representatives from the City of Toronto. In addition to our regular contacts with these partners, we also consulted with other stakeholders (such as data stewards and potential end-users of the toolkit). We sought feedback on our outputs throughout the development of the toolkit and continue to work closely with our partners as we operationalize the toolkit.

The first step in developing the quantitative workforce planning tool was a comprehensive environmental scan of data sources and elements that could be used to inform health workforce planning in the City of Toronto. While wide-ranging, our scan was not exhaustive; its goal was to identify exemplars of data that could be considered for use in quantitative workforce planning models.

We identified sources of data related to the population of patients using primary care services in the City of Toronto, including:

- The Census of Population, the National Household Survey, and the Canadian Community Health Survey, each managed by Statistics Canada;
- The City of Toronto Open Data Catalogue; and
- The Ontario Community Health Profiles Partnership.

Various data elements were examined, including variables related to demography, socioeconomic and cultural diversity, health status, health services utilization and needs, and metrics of unmet healthcare need. We specifically sought population variables (such as gender, language, race, Indigenous identity, and disability) that would facilitate the addition of a health equity dimension to workforce planning activities in the City of Toronto.

We also identified sources of data related to the health workforce in the City of Toronto. These sources included health workforce data from:

- The Toronto Central LHIN Health Provider Census;
- The Health Professions Database, held by the Ontario Ministry of Health and Long-Term Care (MOHLTC);
- The Ontario Physician Resource Data Centre (OPHRDC);
- The Institute for Clinical Evaluative Sciences (IC/ES), a not-for-profit research institute that maintains an array of Ontario’s health-related data; and
- The College of Nurses of Ontario (CNO), the Canadian Federation of Nurses Unions (CFNU), the Canadian Association of Schools of Nursing (CASN), and the Canadian Council of Registered Nurse Regulators (CCRNR).

We examined data elements related to the stock of providers, their activity and participation rates, their scopes of practice, inflows to and outflows from the workforce, provider practice patterns, and productivity. As with our scan of population data, we specifically sought workforce variables—such as gender, race, Indigenous identity, and language—that would bolster the capacity of the Toronto Region to conduct workforce planning with a health equity dimension.

We used three criteria to assess the datasets and data elements identified in the scan: *quality*, *availability*, and *comprehensiveness*. For each criterion, we elaborated an approach based on principles of sound health workforce planning to help the Toronto Region understand the suitability of the data for use in planning. These considerations were intended to guide decisions about the pursuit of specific data sources and about the inclusion or exclusion of specific data elements in the health workforce planning process.

- *Quality* The highest quality data should be chosen to populate the model, with due consideration of the source, strengths, and limitations of each variable. For example, both Census and National Household Survey data undergo a rigorous and transparent quality assessment process [4]. In some cases, as a result of non-response bias, estimates from the National Household Survey may be of inferior quality to those from the Census. These differences should be considered when evaluating the suitability of a given variable from a given source for inclusion in modelling. If high-quality data are not available, we suggested that planning strategies other than quantitative modelling be considered.
- *Availability* Priority should be given to data that are readily available. These include data in the public domain as well as those datasets curated by the Toronto Region. Custom data requests should be assessed for feasibility in terms of timely access to data, comparability and linkability of data, and cost.
- *Comprehensiveness* Selected datasets should facilitate the analysis of a wide range of scenarios. Datasets should be as comprehensive as possible, incorporating diverse factors related to both the population and the health workforce. Data should be as current as possible, with historical data included when available. Data should be comprehensive enough to allow for analysis at multiple geographies—including the neighbourhood, the sub-region, and the entire City of Toronto levels—depending on the scenario in question. Not all data will be necessary for all scenarios, but datasets may be included with a view to providing contextual information or contributing to future evaluation of policy decisions.

We also reviewed population grouping methodologies, including the Canadian Institute for Health Information (CIHI) Population Grouping Methodology [5] and the Johns Hopkins ACG (Adjusted Clinical Groups) System [6], with a view to identifying the methodology most suitable for use by the Toronto Region in a quantitative workforce planning model. Both the CIHI Population Grouping Methodology and the Johns Hopkins ACG System use clinical and demographic profiles to assign patients to clinically coherent case-mix groups. The CIHI Population Grouping Methodology also includes a tool, based on Canadian data, that connects population health characteristics with requirements for health services. The tool predicts the need for visits to a family physician, the anticipated number of Emergency Department visits, and the probability of admission to long-term care.

Our scan and assessment of data elements informed the development of a fit-for-purpose conceptual model ready to be populated with data. Data availability also informed the refinement of the model and the addition of additional modules that respond to scenarios of interest. Close collaboration and regular consultation with the Toronto Region throughout the process served to broaden their understanding of the data available for planning and of the implications of data quality, availability, and comprehensiveness on the quantitative modelling process.

## Results

### Data scan and quality assessment

#### Population

Many comprehensive, high-quality data on population health characteristics are available for use in population needs-based health workforce planning, though most (if not all) are not designed explicitly for these purposes. The results of the scan for data related to population demography, cultural and socioeconomic diversity, population health status, and health service utilization are shown in Tables 1, 2, and 3.

**Table 1:** Population demography, cultural, and socioeconomic diversity

Population profile data	Geographies	Variables	Source	Years	Quality
Demography	Neighbourhood Sub-region LHIN	Age Gender	Census	1996, 2001, 2006, 2011, 2016, Projections 2016–2041	High
Cultural and socioeconomic diversity	LHIN Neighbourhood	Language	Census	1996, 2001, 2006, 2011, 2016	High
	Neighbourhood	Immigration	Census	1996, 2001, 2006, 2016	High
			NHS	2011	Moderate
	Neighbourhood	Visible minority status	Census	1996, 2001, 2006	High
			NHS	2011	Moderate
	Neighbourhood	Education	Census	2006, 2016	High
			NHS	2011	Moderate
	Neighbourhood	Employment	Census	2006, 2016	High
			NHS	2011	Moderate
	Neighbourhood	Income	Census	1996, 2001, 2006, 2016	High
			NHS	2011	Moderate
	Neighbourhood	Social assistance	Toronto Employment and Social Services, Data Mart	2008, 2012	
	Neighbourhood	Equity	Census, Urban HEART	2008	High
	Neighbourhood	Child development	City of Toronto	2008, 2011, 2015	High

**Table 2:** Population health status

Population profile data	Disease condition	Variable	Geographies	Years	Source	Quality
Health status	Diabetes Asthma Hypertension Mental health and addiction-related visits COPD	Prevalence	Neighbourhood	2015, 2012, 2011, 2007, 2001–2003	Ontario health profiles	High
			Sub-region	2015		
			LHIN	2015, 2012, 2007		
			Non-resident			
	Chlamydia Gonorrhoea		Neighbourhood	2008–2012	Ontario health profiles	High
	Breast cancer	Screening	Neighbourhood	2013/2014–2014/2015, 2009–2011	Ontario health profiles	High
			Sub-region	2013/2014–2014/2015		
			LHIN	2013/2014–2014/2015, 2009–2011		
	Cervical cancer	Screening	Neighbourhood	2012/2013–2014/2015, 2008–2011	Ontario health profiles	High
			Sub-region	2013/2014–2014/2015		
			LHIN	2013/2014–2014/2015, 2009–2011		
	Colorectal cancer	Screening	Neighbourhood Sub-region LHIN	2015, 2011	Ontario health profiles	High
	Eye exams among people with diabetes		Neighbourhood	2006, 2010–2012	Ontario health profiles	High

**Table 3:** Population health services utilization

Population profile data	Geographies	Years	Source	Quality
Interprofessional care	Neighbourhood	2016	Ontario health profiles	High
Enrolment and continuity	Neighbourhood Sub-region LHIN	2011–2013	Ontario health profiles	High
Preventable hospitalizations	Neighbourhood LHIN	2012–2014, 2014–2016	Ontario health profiles	High

Multiple sources of data are available to the Toronto Region to support planning. The information is available at various levels of geography and for a variety of time periods.

We identified several populations for which data are not available, or the available data are not of sufficient quality, to allow for quantitative modelling. These populations include Indigenous, homeless, and non-insured persons. For these populations, we suggested that other sources of data—such as local surveys—be considered and that decision-makers advocate for better data to support planning for equitable provision of healthcare for these groups of patients.

#### Workforce

Some high-quality data on the health workforce are available for use in health workforce planning. The results of the scan for data related to physicians, nurses, nurse practitioners, and other regulated health professionals, including those working in two inter-professional primary care delivery models operating in Ontario, are shown in Table 4.

**Table 4:** Health workforce data

Health service provider	Variables	Years	Source	Quality
Physicians	Demographic, geographic, specialty, practice	1992–present	ICES: IPDB, CPDB	High
Nurses	Membership statistics	2016, 2017	CNO	High
	LHIN regional summaries	2016	CNO	High
Nurse practitioners	Graduates	2016	CASN	High
	Practice analysis	2015	CCRNR	High
Regulated health professionals*	59-element minimum dataset**	2008–present	MOHLTC: Health Professions Database	High
Family health teams	MD, NP, RN, RPN, Mental Health Worker, Educator, Pharmacist, RD, Social Worker	2016	ICES	High
Community health centres	Controlled dataset—accessible by custom request		ICES	High

*Audiologists, Chiropodists/Podiatrists, Chiropractors, Dental Hygienists, Dental Technologists, Dentists, Denturists, Dieticians, Kinesiologists, Massage Therapists, Medical Laboratory Technologists, Medical Radiation Technologists, Midwives, Nurse Practitioners, Occupational Therapists, Opticians, Optometrists, Pharmacists, Pharmacy Technicians, Physiotherapists, Psychologists, Registered Nurses, Registered Practical Nurses, Respiratory Therapists, Speech-Language Pathologists, Traditional Chinese Medicine Practitioners and Acupuncturists

**Minimum dataset elements: demographics, language of care, registration status and class, postal code of residence, geographic history of registration and practice, specialty certification, education within and outside the profession, employment history, current employment, practice activities, practice characteristics

Data related to the health workforce are comprehensive, generally of high quality and may be accessed by regional planners through an application and approval process. Capturing practice activities of different health worker cadres is more challenging, and quantitative data on service provision, and on where practice activities overlap between professions, are less robust.

#### Population grouping methodology

We assessed the CIHI Population Grouping Methodology [4] as the most promising method to project needs-based primary care service requirements within the City of Toronto. This innovative, made-in-Canada grouping methodology uses individual-level clinical and demographic data to estimate population health needs, including the predicted number of visits to a family physician (Table 5). Application of this methodology to a region, such as the City of Toronto, with plentiful neighbourhood-level data on population health characteristics, including the social determinants of health, would allow decision-makers to develop a thorough understanding of the influence of various local population characteristics on the need for physician services, and to plan accordingly.

**Table 5:** CIHI population grouping methodology

Inputs		Outputs
Demographic	Registered persons database	Health profile group Predicted healthcare costs Predicted use of selected health system resources: ∙ Number of visits to a family physician ∙ Number of Emergency Department visits ∙ Probability of admission to Long-Term Care
Clinical	Ontario Health Insurance Plan (OHIP) Claims Database Discharge Abstract Database National Ambulatory Care Reporting System Continuing Care Reporting System	

### Model development

**Fig. 3:**
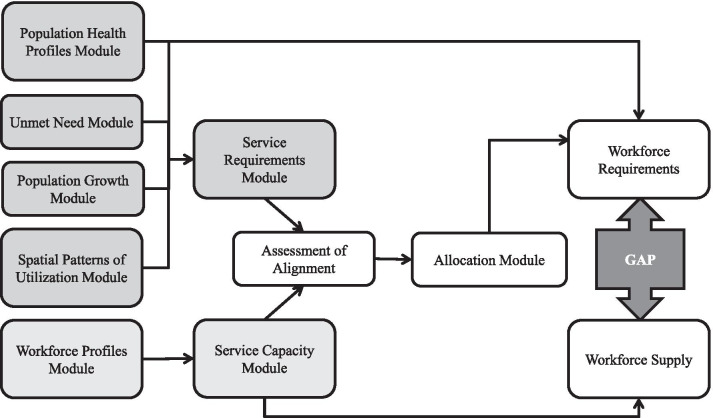
Primary care health workforce planning model

Embedded within the health workforce planning toolkit developed for the Toronto Region is a quantitative workforce planning model (Fig. 3). Using data elements related to population health characteristics and health workforce profiles, as well as the CIHI Population Grouping Methodology, the model examines the alignment of the service requirements of the population with the service capacity of the workforce. The model consists of a series of modules that capture data to address specific issues that are relevant to primary care planning in the region. Scenarios can be introduced at key decision-points in the model to assess the impact of changing population health profiles, changing workforce profiles, changing context, and alternate models of care. Data sources supporting scenario analyses may be descriptive and of either a quantitative or qualitative nature.

#### Step 1: estimation of population service requirements

Four modules related to the population of the City of Toronto contribute to the estimation of population service requirements. These modules capture neighbourhood-level information about population health, spatial patterns of utilization, unmet need, and population growth. Each module, on its own, enhances our understanding of the context and factors that impact primary care service requirements in the City of Toronto. The modules also articulate with the Service Requirements Module, either by contributing a term to the equation describing service requirements, or by facilitating the calculation of future service requirements.

The Population Health Profiles Module summarizes information about population characteristics at a neighbourhood level. These data include the age and income distribution of the population, sociodemographic characteristics and marginalization indices, disease prevalence, and specific measures of health services utilization. Detailed profiles of the population of each neighbourhood can be generated, allowing the Toronto Region and other partners to better understand the factors that impact the need for primary care services at a local level.

Patient mobility was identified by the Toronto Region as a unique regional planning challenge. The Spatial Patterns of Utilization Module characterizes patterns of care-seeking across the city. A snapshot of where patients receive care is captured in a matrix that allows the Toronto Region to identify patterns in care-seeking and adjust estimates of service requirements on the basis of where patients from specific neighbourhoods are likely to seek care. The module also captures utilization of primary care services by patients from outside the City of Toronto. Considering spatial patterns of utilization allows Toronto Region to plan to provide services to the patients who reside within the City of Toronto, while also acknowledging the realities that patients can choose where to receive their care and that many patients travel from outside the city to access care in Toronto. The Spatial Patterns of Utilization Module distributes care needs across the city according to a baseline scenario anchored in current patterns of care-seeking. Given that distortions in spatial patterns of utilization may arise as a result of limited supply constraining care-seeking, or of supplier-induced demand, for example, this module allows the Toronto Region to explore simulated alternative scenarios that redistribute needs. By embedding the capability to analyse various alternate scenarios—such as a greater proportion of care accessed closer to home—the model acknowledges and addresses inefficiencies in current patterns of care-seeking resulting from misalignments in the current system.

Unmet healthcare needs are an important contributor to service requirements, and estimates of service requirements are strengthened by explicit consideration of these needs. As such, the Unmet Need Module synthesizes information related to potentially unmet need for primary healthcare. For example, the module captures data pertaining to low urgency Emergency Department visits and hospitalizations for Ambulatory Care Sensitive Conditions. Data from the Canadian Community Health Survey, as well as consultations with primary care providers in the community, can also contribute to a well-rounded understanding of community-level unmet need. Using quantitative data and descriptive data from local consultations, a subjective measure of unmet primary healthcare need can be derived and added to the calculation of estimated service requirements.

Another priority for the Toronto Region was to characterize the impact of population growth on the need for primary care services. The Population Growth Module synthesizes neighbourhood-level estimates of population growth due to vertical development produced by the City of Toronto Planning Department. This module allows the Toronto Region to identify population growth “hot-spots” and to produce estimates of future service requirements that are adjusted for anticipated population growth.

The estimation of service requirements is conducted for a defined geographic planning region. The model is designed to produce estimates of service requirements for single neighbourhoods; these neighbourhood-level estimates can then be aggregated to produce estimates of service requirements for a group of neighbourhoods, a sub-region, or the entire City of Toronto.

Service requirements are defined as the number of primary care visits needed for patients receiving care within the neighbourhood. These patients may reside in the neighbourhood in question, in another neighbourhood in the City, or outside of the City. Service requirements are derived using the CIHI Population Grouping Methodology, which produces individual-level outputs—predicted number of visits to a family physician in the next year—that are then aggregated to the population level and adjusted for contextual factors such as patterns of care-seeking.

In a given year, in a given neighbourhood (N_*x*_), the estimate of service requirements is described by the following equation:

$$\begin{gathered} {\text{V\_TOT\_N}}_{x} = \left( {{\text{V\_RES\_N}}_{x} } \right)\left( {{\text{P\_RES\_N}}_{x} } \right) \, \hfill \\ \,\,\,\,\,\,\,\,\,\,\,\,\,\,\,\,\,\,\,\,\,\,\,\,\,\,\, + \sum {\left[ {\left( {{\text{V\_NRES\_N}}_{n} } \right)\left( {{\text{P\_NRES\_N}}_{n} } \right)\left\{ {n = 1 - 140|n \ne x} \right\}} \right]} \hfill \\ \,\,\,\,\,\,\,\,\,\,\,\,\,\,\,\,\,\,\,\,\,\,\,\,\,\, + {\text{ V\_NCITY}} + {\text{UMN,}} \hfill \\ \end{gathered}$$

where in neighbourhood *x*: V_TOT_N_*x*_ is the total number of visits required in neighbourhood *x*; V_RES_N_*x*_ is the number of visits required by residents of neighbourhood *x*; P_RES_N_*x*_ is the proportion of primary care visits that residents of neighbourhood *x* receive in neighbourhood *x*; V_NRES_N_*n*_ is the number of visits required by residents of neighbourhood N_*n*_, where *n* ranges from 1 to 140 but does not include *x*; P_NRES_N_*n*_ is the proportion of primary care visits residents of neighbourhood N_*n*_ receive in neighbourhood *x*, where *n* ranges from 1 to 140 but does not include *x*; V_NCITY is the number of visits received in neighbourhood *x* by patients residing outside the City of Toronto; and UMN is a measure of unmet need.

#### Step 2: estimation of workforce service capacity

Estimation of the service capacity of the workforce of each included profession is conducted independently and begins with the identification of the stock of providers available to provide service. Adjustments can then be applied to account for each factor that influences the service capacity of the workforce. These factors include inflows (immigration), outflows (emigration), and attrition due to death and retirement. Additional adjustments can be applied to account for activity rates, participation in the provision of care within the City of Toronto, and scope of practice that varies between providers. More detailed adjustments, such as an adjustment to account for changing practice patterns, can be applied, subject to available data. Finally, a subjective productivity variable allows for scenarios related to productivity to be incorporated into the model, adjusting the workforce service capacity according to productivity assumptions.

The end result of these calculations is an estimate of the total service capacity of the workforce. Due to differences in the data elements available within current datasets, the units of analysis vary. The unit of analysis for physician service capacity is *visits* per year, while for other regulated primary healthcare professionals, the unit of analysis is *hours* per year. The unit of analysis of activity for Nurse Practitioners may be either *visits* or *hours* per year, depending on the dataset employed. These estimates are integrated prior to proceeding further in the model.

#### Step 3: assessment of alignment between service requirements and service capacity

The next step in the model is an assessment of the alignment between service requirements and service capacity. In the case of perfect alignment, no further action is necessary. It is more likely, however, that a gap exists between the requirements of the population for healthcare services and the capacity of the workforce to deliver these services. In this case, an allocation process is suggested to explore ways of minimizing this gap.

#### Step 4: allocation of services across providers and models of care

The goal of this step in the planning process is to optimize the distribution of services, such that service capacity approximates service requirements as closely as possible. This exercise is an important but often overlooked part of planning. By including this step, our model embeds a systematic approach to minimizing the gap between workforce requirements and supply, and optimizing the alignment of the health workforce with the needs of the population.

Allocation may be conducted quantitatively, qualitatively (descriptively), or both. The leading practice quantitative allocation methodology uses a ‘plasticity matrix’ [7]. This methodology compares actual workforce activities with a standard or benchmark activity distribution to examine the sufficiency of the existing workforce, accounting for flexible and overlapping scopes of practice within and between professions and specialties. The methodology enables the shifting of services within a given specialty or between specialties or professions to achieve an optimal distribution of services. It is important to note that task shifting can only be accomplished between professionals where regulated scopes of practice overlap.

The descriptive allocation process included in this planning toolkit is inspired by adjusted service target-based planning [8–11]. The process estimates the requirements of a specific population for a defined package of primary care services. Services can then be iteratively allocated to providers with relevant scopes of practice until optimal alignment between service requirements and service capacity is achieved. This approach accounts for factors that are often at play in health systems, including skill mix within the workforce, current or projected workforce availability, service costs, and emerging models of care.

In the context of primary care services in the Toronto Region, shifting visits between family physicians and nurse practitioners presents an opportunity to optimize allocation of services (and the scope of practice of each). In order to accomplish this allocation using a plasticity matrix, detailed quantitative data regarding the scopes of practice of both family physicians and nurse practitioners, and an understanding of where these scopes overlap and where they differ, are necessary. Unfortunately, quantitative data of this sort are not of high enough quality and sufficient detail at this time. As such, a descriptive allocation process can be undertaken locally, with the goal of defining unique and overlapping scopes of practice. Services that are shared between professions can be shifted in order to minimize the gap between workforce requirements and workforce supply. This task shifting step can optimize skill mix within the workforce, and facilitates all providers practising to the full extent of their scopes of practice. This step can be integrated into a scenario analysis at the allocation phase.

Alternatively, shifting primary care visits between in-person and virtual care presents another opportunity to optimize alignment. Because virtual models of care are relatively new in Ontario and systems to support them are still evolving, descriptive data and close consultation with local providers will guide the process of allocation of visits to virtual care.

#### Step 5: final assessment of alignment between workforce requirements and workforce supply

The gap between workforce requirements and workforce supply offers important insights into the state of the local health system and can be helpful in informing policy development. If requirements exceed supply in a defined geographic planning region, then plans can be made to supplement existing resources to better meet the needs of the population. Conversely, if workforce supply exceeds workforce requirements, then resources can be diverted to other areas experiencing greater need. In a system with limited resources, such analyses are foundational to effective and equitable distribution of healthcare resources. Local health leaders, primary care providers, and patients themselves can validate the gap analysis, indicate whether the results resonate with their experiences, and provide local intelligence to guide the planning process and offer potential solutions to local health service issues.

### Scenario analyses

As described by Chamberland-Rowe et al. (part 1) [2], scenario analyses are designed to simulate the potential implications of changes that could occur within the system. Policy-makers can test the impact of different scenarios, at different points in the model, such as emerging population characteristics, changing workforce profiles, fluctuating population growth, evolving spatial patterns of utilization, increased or decreased unmet need, or alternate models of care. Altering the value of various parameters within the model and examining the results facilitates decision-making that accounts for a range of possible futures. Scenario analyses may be supported by quantitative or descriptive data and provide an opportunity to engage stakeholders and incorporate local intelligence into decision-making, thereby promoting greater understanding and acceptance of planning and resource allocation decisions.

## Discussion

The toolkit is a fit-for-purpose collection of descriptive and quantitative processes to guide and support the Toronto Region in conducting integrated primary care workforce planning. By assembling a selection of multi-methodological tools, and providing guidelines and support for their use, we have established a transparent and accessible approach that can help to make the complexities of health workforce planning more manageable for a regional health authority.

Addressing the specific objectives and planning needs of the Toronto Region, the quantitative model is comprehensive and explicitly considers a multiplicity of factors influencing both service requirements and service capacity. The modular approach allows the Toronto Region to focus on specific issues that are relevant to regional planning. To the extent permitted by the availability of high quality data, these factors are considered quantitatively; when data are not available, a detailed descriptive modelling process is offered.

Our approach is consistent with extant population needs-based health workforce planning models. In particular, our scan for data sources to support the calculation of population service requirements yielded results very similar to the three data inputs—population, health status, and level of service—characterized by Tomblin Murphy et al. [12].

The model we developed is foundational and versatile; expansion of the model through additional modules (when data become available) will yield a more complex model capable of addressing more complex scenarios. This modular approach also facilitates the interfacing of primary care planning with other health workforce planning efforts within the broader health system.

While the data sources we identified are specific to the City of Toronto, and the toolkit explicitly addresses the unique needs and priorities of the Toronto Region, the planning processes we describe are eminently transferrable. The principles are adaptable to other contexts and jurisdictions and represent a robust and dynamic collection of tools for iterative, integrated, needs-based health workforce planning.

Our approach prioritizes engagement with system stakeholders and builds local health workforce planning capacity. Although the operationalization of the planning processes and model is still in progress, it has become clear that the collaboration has resulted in a strong commitment to planning. We have observed significant investment in the process and outcomes on the part of the staff and leadership at the Toronto Region, data stewards, local primary care providers, and other stakeholders in the City of Toronto.

## Challenges

One of the first challenges we encountered was in defining primary care and identifying which professions and services should be included in primary care planning activities within the City of Toronto. Recognizing that primary care can vary in different contexts, we ultimately took an inclusive and pragmatic approach, including data sources on all professions and services that could possibly be of value in planning. The modular nature of the toolkit allows those primary care providers and primary care tasks most relevant to a given planning exercise to be included when necessary.

A second challenge, common to many jurisdictions in Canada and abroad, was data availability. We identified currently available sources of high quality data to populate the quantitative model. However, we also identified significant data gaps related to certain populations, professions, and professional activities.

Finally, we experienced challenges related to health system transformation. A new government has introduced a programme of health system reform and the role of regional agencies with respect to health workforce planning is evolving. The Toronto Region continues to move forward with planning, but the transformation resulted in considerable uncertainty and led to turnover of staff and delays in data access.

## Limitations

One notable difference between our quantitative model and that of Tomblin Murphy et al. [12] is that ours lacks a module related to training. The Toronto Region has no policy levers related to training and is not able to make decisions related to health professional education. As such, our model assumes that the health workforce in the City of Toronto is not limited in any way by the training pathway. This assumption, while arguably reasonable for Toronto, may not be applicable in other jurisdictions, particularly those more rural or underserved.

A recognized limitation of the CIHI Population Grouping Methodology is that it does not yet capture other primary care providers beyond family physicians. As a result, our ability to use quantitative tools to model the need for primary care services delivered by providers other than physicians is currently limited.

## Conclusions

The objective of health workforce planning is not to predict the future, but instead to allow policy-makers to understand how various factors interact to influence the population need for healthcare services and the ability of the workforce to meet this need. We have assembled a fit-for-purpose toolkit—including a quantitative model—to support integrated, multi-professional, needs-based primary care workforce planning. The toolkit and model enable the Toronto Region to understand their health workforce and the population they serve, to assess the alignment between service requirements and service capacity, and to generate scenarios with regional relevance. The approach supports the Toronto Region in making evidence-informed policy and planning decisions for equitable delivery of regional primary care services.

## Data Availability

Data sharing is not applicable to this article as no datasets were generated or analysed.

## Abbreviations

ACG: Adjusted Clinical Groups
CASN: Canadian Association of Schools of Nursing
CCRNR: Canadian Council of Registered Nurse Regulators
CFNU: Canadian Federation of Nurses Unions
CHWN: Canadian Health Workforce Network
CIHI: Canadian Institute for Health Information
CNO: College of Nurses of Ontario
HWP: Health Workforce Planning
IC/ES: Institute for Clinical Evaluative Sciences
LHIN: Local Health Integration Network
MOHLTC: Ministry of Health and Long-Term Care
OPHRDC: Ontario Physician Human Resources Data Centre
